# The ability to predict actions of others from distributed cues is still developing in 6- to 8-year-old children

**DOI:** 10.1167/jov.21.5.14

**Published:** 2021-05-17

**Authors:** Emalie McMahon, Daniel Kim, Samuel A. Mehr, Ken Nakayama, Elizabeth S. Spelke, Maryam Vaziri-Pashkam

**Affiliations:** 1 Department of Cognitive Science, Johns Hopkins University, Baltimore, MD, USA; 2 Department of Hearing and Speech Sciences, Vanderbilt University, Nashville, TN, USA; 3 Department of Psychology, Harvard University, Cambridge, MA, USA; 4 Data Science Initiative, Harvard University, Cambridge, MA, USA; 5 Department of Psychology, Harvard University, Cambridge, MA, USA; 6 Department of Psychology, Harvard University, Cambridge, MA, USA; 7 Laboratory of Brain and Cognition, National Institute of Mental Health, Bethesda, MD, USA; 8 School of Psychology, Victoria University of Wellington, Wellington, New Zealand

**Keywords:** action prediction, development, action understanding, social interaction, biological motion

## Abstract

Adults use distributed cues in the bodies of others to predict and counter their actions. To investigate the development of this ability, we had adults and 6- to 8-year-old children play a competitive game with a confederate who reached toward one of two targets. Child and adult participants, who sat across from the confederate, attempted to beat the confederate to the target by touching it before the confederate did. Adults used cues distributed through the head, shoulders, torso, and arms to predict the reaching actions. Children, in contrast, used cues in the arms and torso, but we did not find any evidence that they could use cues in the head or shoulders to predict the actions. These results provide evidence for a change in the ability to respond rapidly to predictive cues to others’ actions from childhood to adulthood. Despite humans’ sensitivity to action goals even in infancy, the ability to read cues from the body for action prediction in rapid interactive settings is still developing in children as old as 6 to 8 years of age.

## Introduction

Walking down a narrow hallway as another person approaches, you have probably encountered the comedy of being unable to anticipate the passerby's direction—you go left, she also goes left; you switch direction only to realize that she has made the same choice. This continues until one of you steps aside. This situation is funny because it has happened to everyone, but nonetheless it happens rarely. Typically, we are reliable predictors of other people's actions.

How do humans succeed at the complex task of anticipating others’ actions? Previous studies have shown that adults predict the target of an action and make anticipatory eye movements to that location (Flanagan & Johansson, 2003). These predictions are likely based on kinematic cues (Cavallo, Koul, Ansuini, Capozzi, & Becchio, 2016; Diaz, Fajen, & Phillips, 2012) that are widely distributed across the body of the actor (McMahon, Zheng, Pereira, Gonzalez, Ungerleider, & Vaziri-Pashkam, 2019; Pesquita, Chapman, & Enns, 2016; Vaziri-Pashkam, Cormiea, & Nakayama, 2017). Adults can use this distributed information to make predictions even when the locus of the information is far from the body part performing the action. For example, when viewing only the head, neck, and shoulders of someone, adults can predict where that person will reach with their finger (Pesquita et al., 2016; Vaziri-Pashkam et al., 2017).

These cues begin early in the actor's movement. For example, adults are able to predict the target of an actor's reach before the actor's finger had even lifted off from the table (McMahon et al., 2019). When the movements prior to the finger's lift-off are occluded, participants are much slower at predicting the target of the reach (Vaziri-Pashkam et al., 2017). These movements that occur prior to the explicit reach are visually subtle and may be the result of postural adjustments preparing the body for a large limb movement (Hodges, Cresswell, Daggfeldt, & Thorstensson, 2000; Hodges, Cresswell, & Thorstensson, 1999). Moreover, adults do not require training to use these subtle preparatory movements for action prediction; naïve adult participants are already experts at predicting the target of another's reach (Vaziri-Pashkam et al., 2017).

Although adults do not seem to improve their action prediction abilities on the time scale of a typical experiment (Vaziri-Pashkam et al., 2017), they may have acquired these abilities through a lifetime of experience predicting the actions of others. Nevertheless, a rich literature shows that these abilities begin to develop in infancy. Infants as young as 5 months of age (Woodward, 1998), and at 3 months under some conditions (Sommerville, Woodward, & Needham, 2005), encode other people's action goals (Woodard, 1998). By 7 months, infants pick up an object that another person has reached for, even if the actor's reach is incomplete and never arrives at the object (Hamlin, Hallinan, & Woodward, 2008). By 11 months, infants who were familiarized with a reaching movement look at the target of an actor's incomplete reach (Cannon & Woodward, 2012).

Over the first year, infants begin to anticipate the goals of another person's action while that action is ongoing. Twelve-month-old infants make anticipatory eye movements toward the target of such an action (Ambrosini, Reddy, de Looper, Costantini, Lopez, & Sinigaglia, 2013; Brandone, Harwitx, Aslin, & Wellman, 2014; Cannon, Woodward, Gredebäck, von Hofsten, & Turek, 2012; Falck-Ytter, Gredebäck, & von Hofsten, 2006; Gredebäck & Kochukhova, 2010; Rosander & von Hofsten, 2011), indicating that they predict action goals. Extending this finding, Geangu, Senna, Croci, and Turati (2015) we found that 6-month-old infants' predictive eye movements are limited to biomechanically plausible actions, and their predictive abilities develop hand-in-hand with their own action capabilities (for a review, see Gredeback & Falck-Ytter, 2015). Thus, infants are able to predict the actions of others, they are sensitive to the kinematics of people's movements, and these two abilities are related.

However, it is an open question whether or not children read the kinematic information in the body of others comparably to adults. Are children also sensitive to subtle preparatory movements, or do they rely on more explicit kinematic cues? Can children use distributed information present through the body of an actor to predict her actions, or do their action predictions depend on attention to the parts of the actor's body that are directly engaged in preforming the action? Differences in the abilities of children and adults would suggest that expertise in action prediction may develop slowly.

To address these open questions, we compared the predictive abilities of 6- to 8-year-old children and adults for the same action task. We used a two-person action paradigm (after Vaziri-Pashkam et al., 2017) in a realistic setting to investigate how children and adults use the kinematics of others’ actions to make predictions. Adults and children played a competitive reaching game. In the game, a confederate (the “Attacker”) sat across from the participant (the “Blocker”) behind a transparent barrier with two targets marked on it. As the Attacker reached as quickly as possible for one of the targets (chosen at random and signaled to the Attacker by a computer via headphones), the Blocker's task was to beat the Attacker to the target, touching the target before the Attacker did.

To determine whether children were able to use distributed information in the body of the Attacker, we occluded different parts of the Attacker's body during the game in different visual conditions. We also created a control condition in which all of the preparatory information was removed. In this condition, the participants played against a dot on a screen rather than an Attacker who was not present. The dot moved on the screen based on the kinematics of the Attacker's finger from the game. By comparing the reaction times of adults and children in each condition with the Attacker to the Moving Dot control condition, we assessed how children and adults utilized their opponent's preparatory movements during action prediction.

## Method

### Participants

To calculate the power for our sample size, we used the smallest effect size (Cohen's *d* = 1.22) from prior work in adults (Vaziri-Pashkam et al., 2017). Our sample of adults (*N* = 13) yielded a within-subject power of 0.98 to detect an effect of size 1.22 with a two-tailed alpha level of 0.05. For the children, we made a conservative assumption that the standard deviation would be twice as large as in adults. Thus, our sample of children (*N* = 23) yielded within-subject power of 0.8 to detect an effect of size 0.61 with a two-tailed alpha level of 0.05.

In total, we recruited 32 children. Four children were noncompliant and were excluded. Five children were excluded from analyses that included the Moving Dot condition due to an equipment failure during the Moving Dot condition. The final sample included 23 children between 6 and 8 years old (*M* = 7.46 years old, *SD* = 0.97 years, 10 females), who were recruited through the Harvard Laboratory for Developmental Studies database. Thirteen adults between 18 and 35 years old (*M* = 24 years old, *SD* = 5 years, eight females) were recruited through the Harvard Psychology Study Pool. All participants were right handed and had normal or corrected-to-normal vision. Participants or participants’ legal guardians gave informed consent prior to participation. Children were rewarded with stickers, and parents were given $5 for travel expenses. Adult participants were compensated $10 for their time. All experiments were approved by the Committee on the Use of Human Subjects in Research at Harvard University.

### Stimuli and procedure

**Figure 1.**
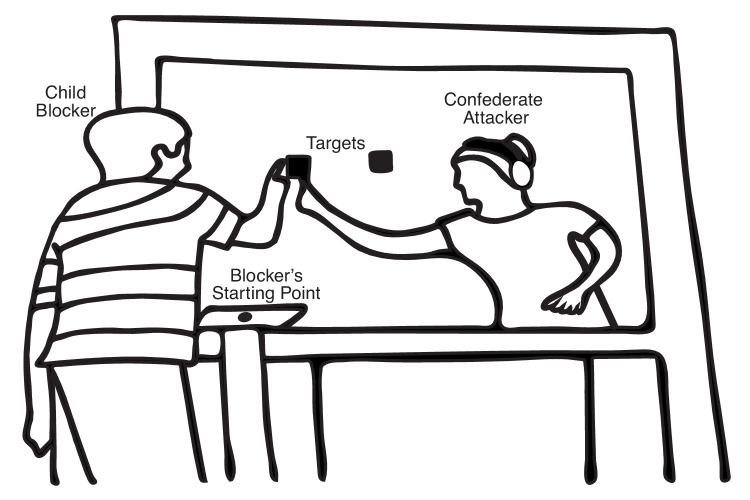
An example illustration of the experimental setup with a child participant in the Full body condition. The child is acting as a Blocker and standing across from a confederate Attacker. The Attacker is signaled through her headphones on each trial to contact either the left or the right target on the Plexiglas screen. The child is told that he should try to beat her to the target. The winner of the trial is the first person to contact the target on that trial. To match height across participants and the confederate, adult participants were seated, but child participants were standing on a platform adjusted to the height of the child.

Participants in this study performed a competitive reaching task similar to our previous studies (McMahon et al., 2019; Vaziri-Pashkam et al., 2017). The participants were always assigned the role of the Blocker. They were positioned across from a confederate Attacker (∼1.2 m apart) and separated by a Plexiglas screen (1.2 m × 1.5 m) on which two foam squares (5 cm) were placed ∼26 cm apart. Adults sat, and children stood on a platform. The platform was raised as needed so that the standing height of each child roughly matched the seated height of the adult confederate (Figure 1).

The Attacker and the Blocker began each trial with their finger on a fixed starting point. The Attacker was then signaled through headphones to reach to either the left or the right target. The Blocker could not hear the instructions. The Blocker was told at the beginning of the session that the goal of the game was to beat the Attacker (i.e., to touch the correct target before the Attacker did). The confederate Attacker reached to the target immediately after hearing the signal. Some of the child participants were reminded of these instructions throughout the experiment.

The confederate and the participant each wore a magnetic sensor on their index finger. These sensors recorded the difference in time between when the Attacker and the Blocker contacted the target. Based on a threshold, the difference in contact time for a given trial determined whether the Attacker or the Blocker won on that trial. Because the Attacker usually won, a threshold was set so that the Attacker and Blocker each won on approximately half of the trials. If the difference was smaller than the threshold, the Blocker won; otherwise, the Attacker won. The threshold for the first five trials was set at a fixed value of 150 ms. After the first five trials, the threshold was set to the median time difference between the Attacker's and the Blocker's contact on all previous trials to ensure an approximately balanced number of wins and losses in each block.

**Figure 2.**
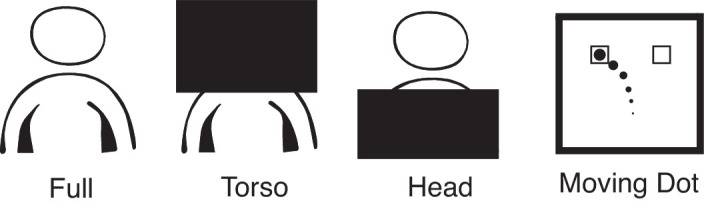
An illustration of the four experimental conditions. In the Full condition, the participants had a full view of the Attacker's body. In the Torso condition, only the torso was visible and the head and shoulders were occluded by attaching black cardboard to the Plexiglas screen. For the Head condition, the head and shoulders were visible, but the torso was occluded. In the Moving Dot condition, the Attacker was replaced with a dot that mapped to the movement of an Attacker's finger.

Participants performed the task over five blocks of 20 trials. The first block was a practice block in which the full body of the Attacker was visible to the Blocker. The next three blocks (counterbalanced across participants) varied among one of three conditions: Full, Torso, or Head (Figure 2). As with the practice trials, in the Full condition, the full body of the Attacker was visible to the Blocker. In the Torso condition, the torso and part of the upper limb of the Attacker were visible to the Blocker, and the head, neck, and shoulders were occluded by attaching a large piece of black cardboard to the Plexiglas screen. In the Head condition, the head, neck, and shoulders of the Attacker were visible to the Blocker, and the torso of the Attacker was occluded by the black cardboard.

A computer monitor (53 cm wide and positioned ∼28 cm behind the Plexiglas screen) replaced the Attacker in the final block for a control condition termed the Moving Dot condition. In this condition, instead of watching the Attacker, participants watched a video of a moving dot, which followed the trajectory from a human Attacker of 20 random trials from a previous study in which an adult Blocker competed against an adult Attacker. This way, the participants only saw the kinematics of an Attacker's finger that was used to control the movements of the dot on the screen, and information from all other parts of the body was removed. The same sensors that recorded the difference in target contact time also recorded the kinematics of the Attacker's finger throughout her movement. The movement of the dot mapped to the finger kinematics so that the dot moved as the Attacker's finger moved during the typical blocks. The *x*- and *y*-positions (left–right and up–down) of the dot on the screen mapped to the *x*- and *y*-positions of the Attacker's finger. Finally, the diameter of the dot mapped to the *z*-position of the finger (distance to the target) such that the dot became larger as the finger moved toward the target. As in the previous blocks, the Blocker's task was to reach as quickly as possible toward the target of the movement of the dot.

### Apparatus

Stimulus generation was done on a Microsoft Windows computer using MATLAB 8.3 (MathWorks) and Psychtoolbox software (Brainard, 1997). A Polhemus Liberty position tracking sensor (1.27 × 2.22 × 1.9 cm) secured to the index fingers of the Attacker and Blocker recorded the three-dimensional position at 240 Hz. For the Moving Dot condition, a Dell monitor (1280 × 960-pixel spatial resolution at 60 Hz; width 53 cm) was used.

### Data analysis and statistics

Analyses were performed in MATLAB and R (R Core Team, 2019) on the kinematic data from the finger sensor. In addition to the standard tools in R, we utilized the ggplot2 (Wickham, 2016), plyr (Wichkam, 2011), and nlme (Pinheiro, Bates, DebRoy, Sarkar, & R Core Team, 2019) libraries. To find the main effect of age group on accuracy and reaction time, we ran two-way mixed-design analyses of variance (ANOVAs). To further investigate the resulting main effects, we ran two-tailed independent or paired-samples *t*-tests (depending on whether the comparison was within or between groups). The resulting *p*-values were controlled for multiple comparison using the false discovery rate method (FDR) (Benjamini & Hochberg, 1995). To control for the effect of the velocity of the Attacker's reaches, we ran a two-way mixed-design ANOVA with movement time added as a factor to determine the main effect of age group and condition after controlling for the reach duration. Finally, in order to investigate the learning effect throughout the experimental blocks in children and adults we used a linear mixed model of reaction time with trial as a fixed factor and subject as a random factor. The data and code are publicly available (https://osf.io/4j5m2/).

## Results

All data analyses are based on the kinematics obtained from sensors on the index fingers of the Attacker and Blocker. Prior to statistical analysis, we calculated the instantaneous velocity at each time point for each trial. The first time point in which the instantaneous velocity of the movement surpassed 15 cm/s was determined to be the starting point of the Attacker's and Blocker's movement. The results of the automated analysis for all trials were manually inspected. If the starting point was determined to be erroneous for a given trial, that trial was removed from all subsequent analyses (7.96% of trials in children and 3.38% of trials in adults). We determined the percentage of trials that were removed per participant and condition and ran a repeated-measures ANOVA. We found no effect of age (*F*(1, 34) = 0.85, *p* = 0.36, η*_p_*^2^ = 0.02) or condition (*F*(3, 102) = 0.97, *p* = 0.41, η*_p_*^2^ = 0.06). Long reaction times over 1 second were also removed from the analysis. These long trials only occurred in children and only accounted for 0.22% of all children's trials.

### Accuracy

A trial was counted as accurate if the Blocker's finger touched the same target as the Attacker. Overall, the accuracy for both children (*M* = 99.1%, *SD* = 2.5%) and adults (*M* = 99.8%, *SD* = 1.1%) was very high. Comparing the accuracies across conditions (Full, Head, Torso, and Moving Dot blocks) and age group (child and adult) using a two-way mixed-design ANOVA, we found no main effect of age (*F*(1, 34) = 2.53, *p* = 0.12, η*_p_*^2^ = 0.07) or condition (*F*(3, 102) = 1.11, *p* = 0.35, η*_p_*^2^ = 0.03) or an interaction between age group and condition (*F*(3, 102) = 0.71, *p* = 0.55, η*_p_*^2^ = 0.02). Similarly, there was no effect of age (*F*(1, 39) = 3.84, *p* = 0.06, η*_p_*^2^ = 0.09) or condition (*F*(2, 78) = 2.87, *p* = 0.06, η*_p_*^2^ = 0.07) or an interaction between age and condition (*F*(2, 78) = 0.78, *p* = 0.46, η*_p_*^2^ = 0.02) after removing the Moving Dot condition and only including the conditions with a human Attacker (Full, Head, and Torso). Thus, children and adults performed similarly in all conditions. Following this analysis, all inaccurate trials were removed from subsequent reaction time analyses.

### Reaction time

**Figure 3.**
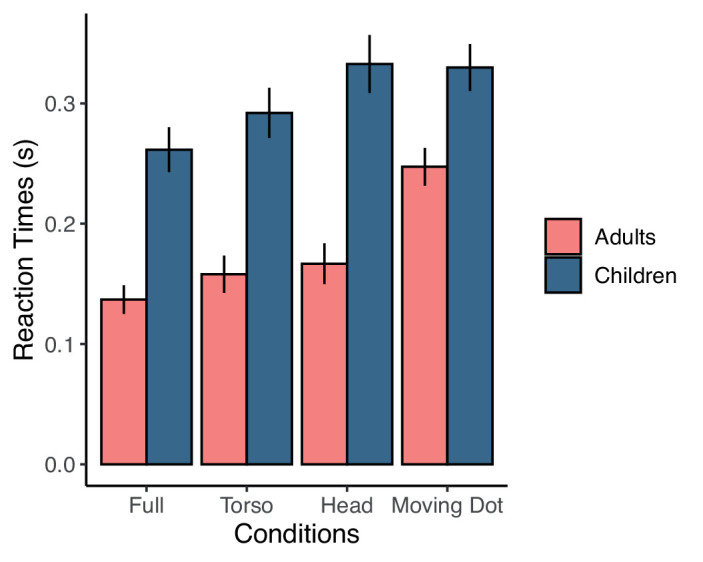
The reaction times of the adults (pink or light gray) and children (blue or dark gray) in each of the four conditions. Children reached more slowly than adults. Error bars indicate the standard error of the mean.

The reaction time of the Blocker was calculated as the difference between the start of the Attacker's movement and the start of the Blocker's movement. As was done for the accuracies, the reaction times of the Blocker were also compared across conditions and age group using a two-way mixed-design ANOVA (Figure 3). We found a main effect of age group (*F*(1, 34) = 57.02, *p* < 0.001, η*_p_*^2^ = 0.63), a main effect of condition (*F*(3, 102) = 62.77, *p* < 0.001, η*_p_*^2^ = 0.65), and a significant interaction between age group and condition (*F*(3, 102) = 8.37, *p* < 0.001, η*_p_*^2^ = 0.20). Thus, children (*M* = 0.30 second, *SD* = 0.07 second) reacted more slowly than adults (*M* = 0.18 second, *SD* = 0.05 second). Moreover, although there was no difference in accuracy among the conditions, adults and children reacted more slowly as conditions varied from full visibility to full occlusion of the body (*M* = 0.21, 0.24, 0.26, and 0.3 seconds; *SD* = 0.07, 0.08, 0.09, and 0.07 seconds for Full, Torso, Head, and Moving Dot conditions, respectively).

There is the possibility that children interpret the movement of the dot differently than adults. To ensure that the differences we observed between children and adults were not due to the Moving Dot condition alone, we ran a separate two-way mixed-design ANOVA for the three conditions in which the participant competed against a confederate Blocker (Full, Torso, and Head conditions). For these conditions, we found a main effect of age group (*F*(1, 39) = 50.63, *p* *<* 0.001, η*_p_*^2^ = 0.56), a main effect of condition (*F*(2, 78) = 50.88, *p* < 0.001, η*_p_*^2^ = 0.57), and a significant interaction between age group and condition (*F*(2, 78) = 6.24, *p* = 0.003, η*_p_*^2^ = 0.14).

We further investigated the pair-wise effects of condition with two-tailed *t*-tests. For adults, all conditions significantly differed from one another (all *t*(12) > 2.69, all corrected *p* < 0.02, *d* > 0.75) except that the torso condition did not differ significantly from the head condition (*t*(12) = 1.01, corrected *p* = 0.33, *d* = 0.28). For children, all conditions differed from one another (all *t*(22) > 3.92, all corrected *p* < 0.001, *d* > 0.82) except that the Moving Dot condition was not significantly different from the Head condition (*t*(22) = 1.03, corrected *p* = 0.32, *d* = 0.21).

We also investigated how children and adults differed in their ability to read information in the body of the Attacker with post hoc ANOVAs. Only including the Head and Torso conditions, we found a main effect of age (*F*(1, 39) = 47.49, corrected *p* < 0.001, η*_p_*^2^ = 0.55) and condition (*F*(1, 39) = 25.76, corrected *p* < 0.001, η*_p_*^2^ = 0.39) and an interaction between age and condition (*F*(1, 39) = 5.88, corrected *p* = 0.03, η*_p_*^2^ = 0.13). Only including the Full and Head conditions, we found a main effect of age (*F*(1, 39) = 52.32, corrected *p* < 0.001, η*_p_*^2^ = 0.57) and condition (*F*(1, 39) = 90.20, corrected *p* < 0.001, η*_p_*^2^ = 0.70) and an interaction between age and condition (*F*(1, 39) = 9.90, corrected *p* = 0.001, η*_p_*^2^ = 0.20). Only including the Full and Torso conditions, we found a main effect of age (*F*(1, 39) = 48.28, corrected *p* < 0.001, η*_p_*^2^ = 0.55) and condition (*F*(1, 39) = 31.88, corrected *p* < 0.001, η*_p_*^2^ = 0.45) but we did not find an interaction between age and condition (*F*(1, 39) = 0.67, corrected *p* = 0.42, η*_p_*^2^ = 0.02). These post hoc ANOVA results reveal that children and adults differ in how they read the information in the Head condition.

To summarize, we found that children and adults differ in their ability to read cues to the target of a reaching action from the body of an opponent. We did not find evidence for a difference in how adults use information in either Head or Torso conditions. However, our findings showed that children are notably slower at reading information in the head and shoulders compared with the torso and arms, where the reach occurs.

Even though the confederates were instructed to act similarly across age groups, it is possible that they might have unintentionally moved more slowly when interacting with children. Unintentional changes in movement, depending on the behavior of opponents in competitive settings, have been demonstrated in previous studies (Naber, Vaziri-Pashkam, & Nakayama, 2013). To determine whether this possible speed variation was the source of the difference between children and adults, we re-ran the main analyses while controlling for the speed of movement. The difference in time between the Attacker's start and her contact with the target (i.e., the movement time) was added as a factor to the ANOVA comparing adults and children and condition. Because the distance between the starting position of the finger and targets was the same in all trials, the movement time can be used as a proxy for velocity.

The confederate Attacker reached more quickly when playing against adults (*M* = 0.17 second, *SD* = 0.01 second) than against children (*M* = 0.20 second, *SD* = 0.03 second, *t*(35) = 4.24, *p* < 0.001, *d* = 1.03). Despite this finding, after including the movement time in the analysis, for all conditions, the main effect of age (*F*(1, 33) = 82.55, *p* < 0.001, η*_p_*^2^ = 0.71) and condition (*F*(3, 101) = 62.73, *p* < 0.001, η*_p_*^2^ = 0.65) and the interaction between age and condition (*F*(3, 101) = 6.35, *p* = 0.02, η*_p_*^2^ = 0.16) did not change qualitatively. We also found a main effect of age (*F*(1, 38) = 16.28, *p* < 0.001, η*_p_*^2^ = 0.65) and condition (*F*(2, 77) = 50.24, *p* < 0.001, η*_p_*^2^ = 0.57) and an interaction between age and condition (*F*(2, 77) = 6.13, *p* < 0.01, η*_p_*^2^ = 0.14) when only the conditions in which the Blocker played against the confederate Attacker were included. This suggests that the Attacker's speed of movement was not the source of difference between the age groups.

### Reaction time advantage

**Figure 4.**
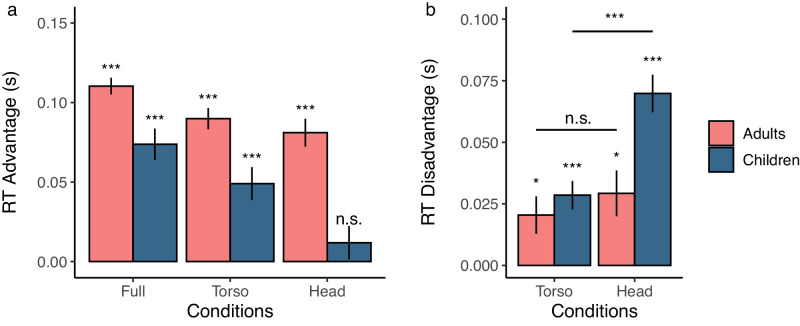
(a) The reaction time advantage of the adults (pink or light gray) and children (blue or dark gray) for the Full, Torso, and Head conditions relative to the Moving Dot condition (not shown). (b) The reaction time disadvantage of the adults and children for the Torso and Head conditions relative to the Full condition (not shown). The tests shown immediately above the bars compare this advantage to zero. Error bars indicate standard error of the mean. (n.s. *p* > 0.05, ^*^p < 0.05, ^***^p < 0.001).

In the previous reaction time analysis, we found that adults were faster than children in all conditions. This finding is not surprising and provides little insight into our main research question, which is whether adults and children differentially read information from the body of the Attacker. Thus, we used the Moving Dot condition as a control condition to account for the baseline differences in the reaction times of children and adults. We computed an “RT advantage” measure by calculating the reaction time advantage that participants gain by having access to information from different body segments (Figure 4a). The RT advantage was calculated by subtracting the reaction time of the Full, Torso, and Head conditions from that in the Moving Dot condition, separately for children and adults. In a two-way mixed-design ANOVA, there was a main effect of age (*F*(1, 34) = 11.32, *p* = 0.002, η*_p_*^2^ = 0.25) and condition (*F*(2, 68) = 41.64, *p* < 0.001, η*_p_*^2^ = 0.55) and an interaction between age and condition (*F*(2, 68) = 4.80, *p* = 0.01, η*_p_*^2^ = 0.12) on the RT advantage.

To further investigate the main effect of condition and age, we compared the reaction time advantage of the adults and children in each condition to zero. This test is the same as if each condition were compared directly to the Moving Dot condition. We found that the reaction time advantage in all conditions was greater than zero (all *t* > 4.30, all corrected *p* < 0.001, *d* > 0.90) except for the Head condition in children (*t*(22) = 1.03, corrected *p* = 0.32, *d* = 0.21). Thus, after accounting for the finding that children are generally slower at the task than adults, we do not find evidence that children are able to pick up on information in the kinematics of the head and shoulders beyond what is available in the finger trajectories alone.

Because our participants came from a large age range, the question might arise whether the oldest children were better than the youngest children at reading information in the head of the Attacker. To answer this question, we performed a regression analysis of the children's ages against their RT advantage in the Head condition. We found an effect of age on the RT advantage (*t*(21) = 2.38, *p* = 0.03, *d* = 0.42). Interpretations of these findings are limited due to the limited age range in the children, but these results suggest that the ability to read the information from the Head of the Attacker develops from 6 to 8 years of age.

### Reaction time disadvantage

To further investigate the difference between Adults and Children in the conditions with a human Attacker, we calculated a RT disadvantage metric. Here, we used the Full condition as the control and looked at the extent to which children and adults were impaired at reading information in the Torso and Head conditions. The repeated-measures ANOVA for the reaction time disadvantage showed a main effect of age (*F*(1, 39) = 6.79, *p* = 0.01, η*_p_*^2^ = 0.15) and condition (*F*(1, 39) = 24.763, *p* *<* 0.001, η*_p_*^2^ = 0.39) and an interaction between age and condition (*F*(1, 39) = 5.88, *p* = 0.02, η*_p_*^2^ = 0.13). Children and adults (all *t* > 2.68, all corrected *p* < 0.02, *d* > 0.75) were disadvantaged in all conditions. However, children had a much greater RT disadvantage for the Head relative to the Torso (*t*(28) = 5.07, *p* < 0.001, *d* = 0.96) but this was not true for adults (*t*(12) = 1.01, *p* = 0.33, *d* = 0.28). Together, these results provide further evidence that children and adults differ in how they read information in the body of the Attacker.

### Learning

**Figure 5.**
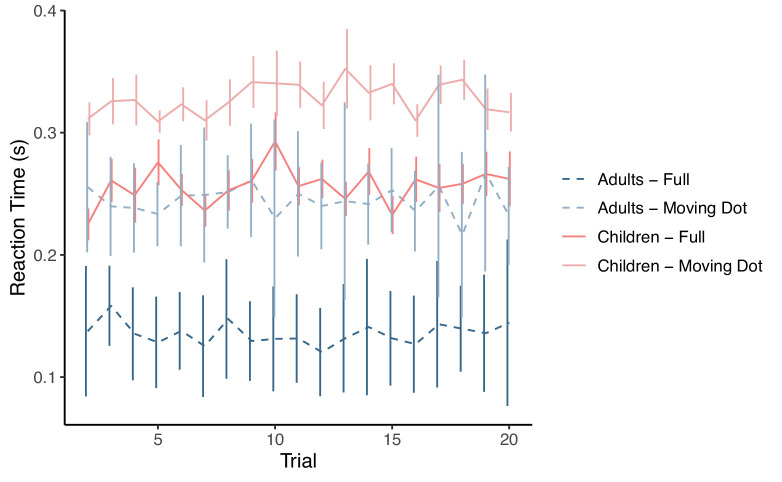
The reaction time for adults and children in the Moving Dot (darker) and Full body (lighter) conditions after the first trial. The reaction times of children (solid) and adults (dashed) did not decrease during a block, suggesting that neither group was learning during the experiment. The Torso and Head conditions are not shown to avoid clutter but had the same pattern (see Results).

To test whether children and adults improved during the experiment, we investigated whether they responded more quickly on the later trials of each experimental block (Figure 5). Each experimental condition was performed in one block and were analyzed separately. The first trial was excluded from the regression analysis due to a novelty effect in all conditions and groups. Using a linear mixed-effects model of reaction time against trial with subject as a random effect, neither adults nor children were found to improve within an experimental block (all *F* < 4.77, all corrected *p* > 0.12). These findings suggest that children showed no short-term improvements in their ability to predict the actions of others during the course of the experiment.

## Discussion

We asked whether 6- to 8-year-old children are able to use kinematic information available in the body of others to predict their goals, and we compared the performance of children with that of adults. Our results suggest that children, like adults, can use subtle, preparatory movements in the body for action prediction. In contrast to adults, however, children seem to rely more heavily on information in the torso and arms (the regions near the origin of action) and less on distant information in the head and shoulders.

In comparing the reaction time of children and adults directly, unsurprisingly, we found that children react more slowly than adults in all conditions. Our primary question of interest was whether children would be able to read preparatory information from the body of the Attacker similarly to adults. Thus, the finding that children predict the actions of others more slowly in a competitive context regardless of the amount of information available is not informative to our central aim.

By occluding large, non-overlapping sections of the Attacker's body, we replicated the previous finding that adults can use distributed information in the body for action prediction (Pesquita et al., 2016; Vaziri-Pashkam et al., 2017). Children were able to use preparatory information, but differed from adults in which parts of the body they relied on most heavily. When only the head, neck, and shoulders were visible, we did not find evidence that children were able to pick up on additional information that was available beyond what was available in a control condition in which only a dot moved on the screen. These results suggest that children particularly rely on information in the lower portion of the body to predict the target of a large reaching action.

The finding that children rely on information in the lower portion of the body to predict the target of a reach may seem contradictory with prior studies finding that even infants as young as 2 months old are able to follow gaze (Scaife & Bruner, 1975). Although it is likely that our child participants were able to follow gaze, we do not think that gaze provides substantial information in this realistic, speeded interactive context. First, prior studies have found that adults are less sensitive to gaze than to kinematic cues (Quesque & Coello, 2014). Second, in a prior occlusion study in similar settings, we found that adults were not substantially impaired when the opponent's eyes were covered (Pesquita et al., 2016; Vaziri-Pashkam et al., 2017). Finally, a study mapping the sources of information in the body of the Attacker in a similar setting found that the eyes did not provide more information about the target direction than movements in the head (McMahon et al., 2019).

Both adults and children have experience viewing and performing reaches, but the experimental game was a novel context for all participants. For this reason, we investigated whether children and adults are able to predict the target of the Attacker's reach more quickly at the end of participation than at the beginning. Replicating prior work in adults (Vaziri-Pashkam et al., 2017), we did not find that to be the case for either children or adults. For these simple actions, adults seem to be experts at predicting common actions without training. Even within our small sample, we found evidence that older children are better able to read information in the upper body of the Attacker like adults, but overall children and adults still differed in how they read information in the body of the Attacker. Thus, although even in infancy children are able to predict actions of adults (Cannon & Woodward, 2012; Woodward, 1998), our current study of action prediction in a realistic context suggests that action prediction may have a prolonged developmental time scale.

Why were children slower at reading information in the head, neck, and shoulders of an actor, when they were relatively good at reading information from the actor's torso, arm, and hand? During a large reaching action, there were larger movements in the lower portion of the body visible in the Torso condition than in the upper portion of the body visible in the Head condition. One possibility is that the visual system of Adults may be more sensitive to the smaller movements of the Attacker's eyes, head, neck, and shoulders than are children. A second possibility is that adults have a more developed cognitive model of human movements that specifies how a distant head movement relates to a concurrent arm movement. Because adults have more motor experience reaching, they also may be better at simulating the future reaching action of a conspecific. This possibility aligns with the direct-matching hypothesis that motor experience for an action is necessary for predicting that action (Cannon et al., 2012; Falck-Ytter et al., 2006; Kanakogi & Itakura, 2011; Kochukhova & Gredebäck, 2010). Note, however, that much of the direct-matching research has focused on whether infants have experience with the motor action at all. In the present study, although adults have more experience reaching, children do also have experience reaching. Ultimately, the current study does not reveal the causes of the developmental change that it documents.

Another possible source of the difference between children and adults is the behavior of the confederate Attackers. Although the confederate Attackers in our study were instructed to reach at the same speed when playing with both adults and children, prior work has shown that, in a competitive context, a competitor may unintentionally reach more slowly if their opponent is slower (Naber et al., 2013). To ensure that the behavior of the Attacker was not the main source of our findings, we controlled for the velocity of the Attacker's reach by adding the velocity of each Attacker to the ANOVA of reaction time by age group and condition. After considering the effects of velocity in this way, all previous findings of differences between age groups and conditions remained. Thus, this analysis alleviates the concern that the Attacker behaving differently toward the two age groups explains our findings.

In our RT advantage analysis, we used the Moving Dot condition as a baseline to account for the overall reaction time differences between children and adults. Several differences existed between the Dot condition and other conditions with the human Attacker. The dot was presented on a 2D screen, whereas the human attacker moved in three-dimensional space. The range of the dot movement was smaller than the space spanned by the finger of the participant (due to the limitations in the size of the computer screen). The dot represents the finger in an abstract way and does not contain a body form. These factors pose potential problems for the use of the Moving Dot condition as a baseline. Despite these differences, it is noteworthy that previous experiments (Vaziri-Pashkam et al., 2017) have shown that the reaction times in the dot condition are comparable to those in a condition in which subjects play against a human attacker with all of the preparatory cues removed. These results suggest that the Moving Dot condition is a reasonable baseline. In addition, the results of the RT disadvantage analysis reveal that the differences between adults and children still persist even after removing the Moving Dot condition and using the Full condition as the baseline.

In our results, we did not find evidence for a difference between the Head and Moving Dot conditions in children. It is still possible that children are able to read information from the head and shoulders, but we were not able to detect a potentially small effect with our sample size. Another caveat is that the Moving Dot moved faster than the attacker in the Head condition. The adult Attackers reached more slowly when playing against children, but the kinematics of the dot were based on an adult Attacker playing against an adult Blocker from a previous study. The faster Moving Dot may have led the children to compensate by reaching faster and, as a result, may have led to the lack of difference between the Moving Dot and the Head condition. Finally, children's impairment in reading cues in the Head condition may be limited to the fast interactive setting of our study. Any of these possibilities could have led to the lack of differences between the Moving Dot and Head conditions in children. Thus, we cannot rule out the possibility that children can read information in the head and shoulders, albeit less efficiently than adults. Future experiments with larger sample sizes and different paradigms can determine if children are able to read cues from the head and shoulders and if the results of our study would generalize to other less demanding tasks.

In conclusion, the current study suggests that, although the adults’ and children's action prediction performance does not improve within an experimental session, prediction shows an improvement from 6 to 8 years of age to adulthood. In order to better understand how the adult visual system has been optimized for prediction, future research should investigate the contributions of visual and motor experience in prediction throughout development.
